# Dental Ageing Offers New Insights Into the First Epigenetic Clock for Common Dolphins ( *Delphinus delphis* )

**DOI:** 10.1002/ece3.72424

**Published:** 2025-11-11

**Authors:** Eva‐Maria F. Hanninger, Katharina J. Peters, Livia Gerber, Ashley Barratclough, Emma L. Betty, Emily I. Palmer, Steve Horvath, Karen A. Stockin

**Affiliations:** ^1^ Cetacean Ecology Research Group, School of Natural Sciences Massey University Auckland New Zealand; ^2^ Marine Vertebrate Ecology Lab, Environmental Futures University of Wollongong Wollongong Australia; ^3^ Securing Antarctica's Environmental Future University of Wollongong Wollongong Australia; ^4^ Australian National Wildlife Collection Commonwealth Scientific and Industrial Research Organisation Canberra Australia; ^5^ National Marine Mammal Foundation San Diego California USA; ^6^ Altos Labs Cambridge UK; ^7^ Epigenetic Clock Development Foundation Torrance California USA

**Keywords:** age biomarkers, chronological age estimation, DNA methylation, life‐history, tooth ageing

## Abstract

Determining exact age in wild odontocetes is essential for understanding population dynamics, survival, and reproduction, yet remains logistically challenging. Although epigenetic ageing is emerging as a valuable approach, only nine species‐specific clocks currently exist. Most have been calibrated using known‐age animals in human care or well‐studied wild populations. Only three previous studies have used dental ages from stranded or bycaught individuals. This is due to concerns that dental age inaccuracies, especially in older animals, may affect epigenetic clock performance. To explore this, we developed the first species‐specific epigenetic clock for common dolphins ( *Delphinus delphis* ), analysing DNA methylation at 37,492 cytosine‐phosphate‐guanine sites in skin samples from stranded and bycaught dolphins with estimated dental ages. Elastic net models with Leave‐One‐Out Cross‐Validation were applied to three subsets: the ‘*relaxed*’ subset (all individuals; *n* = 75, median absolute error (MAE) = 2.02, *r* = 0.81, *R* ^2^ = 0.66), the ‘*strict*’ subset (excluding individuals with minimum dental age estimates only; *n* = 73, MAE = 2.29, *r* = 0.81, *R* ^2^ = 0.66), and the ‘*restricted*’ subset (excluding outliers with prediction errors > 6 years; *n* = 63, MAE = 1.80, *r* = 0.91, *R* ^2^ = 0.82) to compare performance. Our models consistently underestimated the age of dolphins > 16 years, even when minimum dental ages were applied, suggesting that absolute errors between dental and epigenetic estimates are unlikely to reflect the dental ageing error. Additionally, post‐mortem decomposition condition code (DCC 1 to 3) did not affect age prediction, signalling promise for future epigenetic clocks calibrated with strandings and bycaught individuals.

## Graphical abstract

We present the first species‐specific epigenetic clock for common dolphins (*Delphinus delphis*), calibrated using dental ages from stranded and bycaught individuals. Despite concerns about dental ageing accuracy, our models showed strong performance and were unaffected by decomposition, supporting the use of post‐mortem samples for epigenetic clock development.

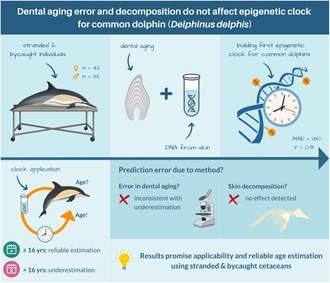

## 1 Introduction

In wildlife management, age determination plays a vital role in understanding population structure, an individual's reproductive state, and lifespan (Barratclough, Sanz‐Requena, et al. 2019; Betty et al. 2022). Such knowledge of population age structure provides crucial insights into population viability and resilience to both anthropogenic and environmental pressures (Betty et al. 2019; Heydenrych et al. 2021; Manlik et al. 2022; Palmer et al. 2022). More broadly, understanding life‐history features is essential for effective conservation and management of protected species (Moore and Read 2008), particularly those impacted by human‐induced threats such as fisheries interactions, habitat degradation, and pollution (Murphy et al. 2018; Peltier et al. 2021; Stockin et al. 2010; Tulloch et al. 2020). However, accurate age estimates are essential to ensure reliable assessment of key life‐history parameters, such as age at sexual maturity (ASM) and inter‐calving intervals (Manlik et al. 2016; Palmer et al. 2022; Verborgh et al. 2021). Yet obtaining such estimates is particularly challenging in odontocetes (toothed whales and dolphins), which exhibit limited external signs of ageing (Barratclough et al. 2023; Beal et al. 2019; Krzyszczyk and Mann 2012). Although total body length can assist in assessing age class (i.e., neonate/juvenile/adult), growth varies greatly among individuals of the same species and age, making it unreliable for precise age estimation (Betty et al. 2019; Chivers 2018). Instead, age estimation requires either long‐term field studies (Connor and Krützen 2015; Peters et al. 2023) or invasive methods, such as tooth extraction (Lockyer 1993; Westgate and Read 2007).

Typically, odontocetes are aged by quantification of growth layer groups in teeth, also known as “tooth ageing” (e.g., Betty et al. 2022; Evans et al. 2002; Lockyer 1993, 1995; Maas 2009; Murphy et al. 2014; Westgate and Read 2007). Tooth ageing requires trained, experienced readers and calibration using individuals of known age (Perrin and Myrick 1980). Although growth layer groups are generally assumed to represent annual deposition in most species—with the exception of beluga whale *Delphinapterus leucas* (e.g., Goren et al. 1987; Waugh et al. 2018)—their interpretation becomes increasingly challenging in older individuals. Contributing factors include varying degrees of tooth wear, compression of growth layer groups, and the presence of accessory lines (additional growth lines that may not correspond to annual deposition; Barratclough et al. 2023; Murphy et al. 2014; Read et al. 2018). Additionally, because of its invasive nature, tooth extraction is typically restricted to post‐mortem investigations, except for procedures conducted under anaesthesia (Barratclough, Wells, et al. 2019), and accordingly does not permit assessment of demography in free‐ranging populations (Betty et al. 2023). Consequently, age estimates are often derived from stranded or bycaught individuals, which may not accurately represent the age structure of free‐ranging populations and may bias population viability analyses and survivorship estimates (Betty et al. 2023).

Emerging molecular methods offer a minimally invasive alternative that can be applied to free‐ranging cetaceans, using samples such as skin or blood, for example, to assess epigenetic patterns of DNA methylation for age estimation (Bocklandt et al. 2011; Horvath 2013; Horvath and Raj 2018; Teschendorff and Horvath 2025). Because of a phenomenon called ‘epigenetic drift’, genomic regions gain or lose cytosine methylation with age (Issa 2014; Poulsen et al. 2007; Sen et al. 2016).

The Mammalian Methylation Consortium carried out epigenome‐wide association studies of age in 348 mammalian species, including mysticetes and odontocetes (Haghani et al. 2023; Lu et al. 2023). The correlation between chronological age and DNA methylation at CpG sites (cytosine‐phosphate‐guanine) enables the development of epigenetic clocks, which are regarded as the most reliable predictors of age available (De Paoli‐Iseppi et al. 2017; Guevara and Lawler 2018; Jylhävä et al. 2017; Simpson and Chandra 2021). The initial development of epigenetic clocks requires a highly accurate known age calibration population (Mayne et al. 2023). To date, 11 epigenetic clocks exist for odontocetes: 3 for the common bottlenose dolphin ( *Tursiops truncatus* ; Barratclough et al. 2021; Beal et al. 2019; Robeck, Fei, Haghani, et al. 2021), 2 for the Indo‐Pacific bottlenose dolphin ( *T. aduncus* ; Peters et al. 2023; Yagi et al. 2024), 1 for the killer whale ( *Orcinus orca* ; Parsons et al. 2023), 1 for the beluga whale (Bors et al. 2021), 1 for Hector's and Māui dolphins ( *Cephalorhynchus hectori hectori* and *C. h. maui*; Hernandez et al. 2023), 1 for Risso's dolphin ( *Grampus griseus* ; Mori et al. 2024), and 2 multi‐species odontocete or cetacean clocks, which were cross‐validated with 9 (Robeck, Fei, Lu, et al. 2021) and 13 species (Zoller et al. 2025).

Most odontocete clocks have been calibrated on animals in human care (Barratclough et al. 2021; Robeck, Fei, Haghani, et al. 2021; Robeck, Fei, Lu, et al. 2021), or long‐term observational studies of well‐monitored populations (Beal et al. 2019; Parsons et al. 2023; Peters et al. 2023; Yagi et al. 2024). At the time of our study, only three prior studies used growth layer groups of stranded and bycaught animals for the initial calibration of their epigenetic clocks (Bors et al. 2021; Hernandez et al. 2023; Mori et al. 2024). Notably, concerns have been raised about whether errors in dental ageing may affect the robustness of epigenetic clocks, particularly in older individuals where growth layer groups can be confused with accessory lines (Bors et al. 2021; Hernandez et al. 2023; Mori et al. 2024; Zoller et al. 2025).

Here, we present the first epigenetic clock for common dolphins (*Delphius delphis*)—an essential tool for future population viability analysis (PVA) in a species highly impacted by human activities (Peltier et al. 2016; Piroddi et al. 2011; Stockin et al. 2009; Tulloch et al. 2020). Further, we provide three distinct models that enhance current understanding of the reliability of dental ageing for age calibration of methylated clocks. Our clock was developed using dental age estimates from individuals spanning the full known lifespan of free‐ranging common dolphins, estimated at approximately 30 years (Perrin 2009), with reports of individuals in human care reaching > 35 years (Murphy et al. 2014). As dental ageing can be less accurate in older individuals because of tooth wear and growth layer group compression, this approach allows us to evaluate how such uncertainties can affect clock accuracy. Specifically, we hypothesised that: (1) a clock model excluding individuals with uncertain dental age estimates would outperform one that includes all samples, and (2) a model on the basis of all samples would perform worse than previously published odontocete clocks calibrated using known‐age individuals from long‐term observational studies or human care.

## 2 Materials and Methods

### 2.1 Sample Collection

As part of ongoing longitudinal studies on health and life history, we accessed a 30‐year tissue archive of aged common dolphins spanning 0 to 34 years of age, for which sex, body length, sexual maturity, reproductive stage, and radiographic bone ageing had been completed a priori. Dental age was assessed via growth layer groups in the dentine of thin, decalcified, and stained sections of teeth by at least two independent, experienced readers over three readings. If no agreement was reached, an additional tooth was prepared and examined by at least two readers until consensus was reached on the age estimate. In total, each animal had 1 to 3 teeth processed for ageing. Age estimates were further validated against additional indicators of physical and sexual maturity, including total body length, the number of ovarian *corpora albicans* scars on the basis of Palmer et al. (2022) and pectoral flipper radiographic ageing (adapted from Barratclough, Sanz‐Requena, et al. 2019). These metrics allowed cross‐verification of age estimates across multiple biological parameters.

To ensure an adequate sampling size for the construction of a reliable epigenetic clock (Mayne et al. 2021), we selected skin from 84 common dolphin samples examined between 2000 and 2023. Sample selection was guided by multiple criteria, including dental age (Table S1) and sex ratio (females: *n* = 47; males: *n* = 37). We included individuals with dental ages spanning the maximum known lifespan of common dolphins, with equal age and sex distribution, as the sample availability would allow. Our samples originated from various mortality contexts, including bycatch (*n* = 9) and strandings (single stranding events: *n* = 46; mass stranding events: *n* = 28), and one individual from human care. Only animals of decomposition condition category DCC 1–3 (Ijsseldijk et al. 2019) were included in the study (Tables S1 and S2).

For 82 common dolphins, exact dental ages were ascertained. For the remaining two samples, only age ranges could be estimated. Here, we applied the minimum year of each age estimate to reduce the risk of age overestimation because of accessory lines. The age range of our samples spanned the estimated lifespan of the common dolphin, with the oldest free‐ranging individual having a minimum dental age of 31 years. Additionally, we included a skin sample from a dolphin in human care (*Kelly*, Napier Marineland, held 1974 to 2008). Because of a pulp anomaly, only an age range could be determined, but Kelly was known to have spent 34 years in human care (Murphy et al. 2014).

### 2.2 DNA Extraction

Genomic DNA was extracted from skin samples using a Quick‐DNA Miniprep Plus Kit (Zymo) following the protocol for solid tissue samples. DNA purity was assessed using a NanoDrop Microvolume Spectrophotometer (Thermo Fisher Scientific) by an automated measurement of absorbance at the wavelengths 260 and 280 nm (Desjardins and Conklin 2010). DNA was considered pure with an absorbance ratio ~1.8 at 260 nm/280 nm (Desjardins and Conklin 2010). DNA was purified using a DNA Clean & Concentrator Kit (Zymo) following the manufacturer's instructions and concentration was measured using a QUBIT 4 fluorometer (Thermo Fisher Scientific) aiming for a total DNA amount per sample of > 250 ng.

### 2.3 DNA Methylation Data

A custom Infinium methylation array (HorvathMammalMethylChip40) assembled with 37,492 CpG sites was used to measure DNA methylation (Arneson et al. 2022). The array targets highly conserved CpG sites across mammals, allowing the assessment of DNA methylation in each mammal (Arneson et al. 2022). Sample quality control followed the Clock Foundation pipeline (Zoller and Horvath 2024). Methylated (M) and Unmethylated (U) channels reporting background signal levels were marked as failed with the *p*‐value threshold of *p* > 0.05. Additionally, the proportion of failed probes was counted for each sample. Samples with more than 1% of failed probes were considered fraction outliers. Further quality control steps included principal component analysis (PCA) performed on the centred, unnormalised beta‐value matrix to identify major sources of variance in the dataset (Mishra et al. 2017), as well as hierarchical clustering analysis, whereas samples with higher than 2 standard deviations from the mean were marked as outliers (Zoller and Horvath 2024). Nine samples were excluded from further analysis (see Results and Table S2).

### 2.4 Subsets

We developed the epigenetic clock using three distinct sample subsets. Details of sample selection for each subset are provided in Table S1, in addition to age distributions by sex (Figure S1).

The first model, referred to as the *relaxed* subset (*n* = 75; age range: 0–34 years) included all samples, also incorporating two individuals for whom only a minimum estimated dental age was available; both originated from individuals > 30 years of age. The second model, termed *strict*, excluded these two individuals and only incorporated animals with confirmed dental ages (*n* = 73; age range: 0–31 years). Following initial analyses, a third model was generated using only samples from the *relaxed* subset that showed an absolute error of less than 6 years between actual and predicted age. This *restricted* subset (*n* = 63; age range: 0–26 years) automatically excluded the two individuals with the minimum age estimates. Except for three samples, all excluded individuals were ≥ 16 years old.

### 2.5 Age Prediction

#### 2.5.1 Elastic Net Regression With LOOCV

To build the epigenetic clocks, we used the MammalMethylClock R package, which incorporates functionalities for the assessment, development, and utilisation of epigenetic clocks (Zoller and Horvath 2024).

The package uses pre‐existing life‐history data from the Animal Ageing and Longevity Database (AnAge^1^). In the case of *Delphinus delphis* , the AnAge data (female average age at attainment of sexual maturity (ASM): 4.8 years, male ASM: 4.3 years) did not align with previously published life‐history data for common dolphins (Danil and Chivers 2007; Murphy and Rogan 2006; Palmer et al. 2022, 2023; Westgate and Read 2007); therefore, we manually overwrote the data with an average value of 8.15, as reported for male and female reproduction in Aotearoa New Zealand common dolphins (female ASM: 7.5 years, male ASM: 8.8 years; Palmer et al. 2022, 2023). For the gestation period, we used 1.05 years, which was determined on the basis of the Aotearoa New Zealand common dolphins (Palmer et al. 2022). On the basis of these life‐history features, we applied a log‐linear transformation to our methylation data (Lu et al. 2023; Zoller and Horvath 2024). This transformation accounts for accelerated epigenetic ageing processes before attainment of sexual maturity (ASM), providing a logarithmic function if age is lower than ASM and a linear form in case age is greater than ASM (Lu et al. 2023). We further used the function fun_llin2.inv., to take species‐specific gestation times into account.

To build a species‐specific epigenetic clock, the package uses an elastic net regression (Zoller and Horvath 2024), which is an algorithm for fitting generalised linear models with elastic‐net penalties (Friedman et al. 2010). The elastic net algorithm works on very large datasets in which the number of predictor variables exceeds the number of observations (Friedman et al. 2010). Elastic nets are particularly useful in situations where there are many correlated predictor variables. We used an elastic net regression model (glmnet package) to determine the optimal mixing parameter (*α*) via cross‐validation on 20 random subsets (each with 2/3 of the data). Models were fit across *α* values from 0 to 1 (in 0.1 steps), and the *α* with the lowest median mean squared error was selected (Barratclough et al. 2021). On the basis of this analysis, the elastic net was chosen (*relaxed* subset: *α* = 0.4, *strict* subset: *α* = 0.5, *restricted* subset: *α* = 0.7) to remove any degeneracies and wild behavior caused by extreme correlations (Friedman et al. 2010; Lu et al. 2023; Zoller and Horvath 2024).

Cross‐validation was completed with a Leave‐One‐Out Cross‐Validation (LOOCV) analysis (Zoller and Horvath 2024). This function uses n‐fold internal cross‐validation, with *n* being the number of samples (Zhang 1993; Zou and Hastie 2005). For each sample of the data, the LOOCV approach skips one sample, fits the epigenetic clock on the remaining data, and predicts the age of the omitted sample (Zhang 1993; Zou and Hastie 2005).

#### 2.5.2 Hybrid Epigenetic Clock

For the *relaxed* subset, we applied a hybrid DNA methylation age prediction approach integrating a random forest classifier (RFC) and two elastic net regression (ENR) models following Barratclough et al. (2024). Physical maturity status was classified using an RFC with 1000 trees, trained to distinguish physically mature dolphins (≥ 18 years for females, ≥ 20 years for males, Palmer 2023). Two ENR models were trained to predict log‐linearly transformed ages, anchored on sex‐specific sexual maturity thresholds (7.5 years for females, 8.8 years for males; Palmer et al. 2022, 2023). The first and second ENR were trained on all individuals and physically mature dolphins, respectively, to refine age predictions in older animals. For both ENRs, the optimal balance between LASSO and Ridge penalties (*α*) and the penalty parameter (*λ*) was selected by nested 5‐fold internal cross‐validation on the training data. Overall model performance was evaluated using 5‐fold cross‐validation. In each fold, the RFC and ENR models were retrained on the training data, and age predictions were generated as a weighted sum of the two ENR models, where the weight for the mature‐only model corresponded to the RFC‐predicted probability of being physically mature. A gestation adjustment of 1.05 years was applied to the final age estimates (Palmer et al. 2022).

#### 2.5.3 Error Metric Assessment

The accuracy of the resulting epigenetic clocks was tested with Pearson correlation coefficients (*r*) and the calculation of median absolute errors (MAEs) between dental and predicted epigenetic age. We further tested the accuracy of the DNA age predictions by performing a linear regression of DNA predicted age against dental age (Barratclough et al. 2021; Beal et al. 2019; Peters et al. 2023). This analysis allowed us to assess how closely the DNA‐predicted ages aligned with dental age estimates. A slope close to 1 and an intercept near 0 would indicate a strong agreement between predicted and observed values, whereas deviations may highlight systematic under‐ or overestimation. The coefficient of determination (*R* ^2^) was also examined to quantify the proportion of variance in chronological age explained by the epigenetic predictions.

#### 2.5.4 Effect of Tissue Decomposition

To assess whether tissue decomposition influenced the accuracy of epigenetic age predictions, we compared the absolute age prediction errors from the *relaxed* subset across decomposition condition categories DCC 1 to 3 (fresh to moderate) assigned post‐mortem during sampling (IJsseldijk et al. 2019). Absolute prediction errors were calculated as the absolute difference between predicted and observed ages for each individual. We then used a Kruskal–Wallis rank sum test to assess differences in error distributions among decomposition condition categories, as the error data did not meet the assumptions of normality.

#### 2.5.5 Effect of Storage Duration

To evaluate whether long‐term storage of samples could bias epigenetic age estimates, storage time was calculated as the difference between the year of DNA methylation analysis (2023) and the year of sample collection, as indicated in the sample identifier. Absolute age prediction errors from across all samples were used as the response variable. Associations between storage time and prediction error were assessed using Spearman rank correlation (robust to deviations from normality) and linear regression, with and without chronological age included as a covariate. For the regression models, residuals were inspected using diagnostic plots and tested for normality using the Shapiro–Wilk test to confirm model assumptions.

#### 2.5.6 Age Predictions on the Basis of Other Odontocetes Clocks

Additionally, our clock's performance was tested in relation to that of other epigenetic clocks, using the MammalMethylClock R package, browsing the Clock database with the search criteria ‘odontocetes’ and ‘skin samples’. We applied previously published multi‐species and species‐specific odontocetes clocks to our samples and compared the respective MAE and *r*‐values between the resulting clocks.

### 2.6 Sex Prediction

To predict the sex of each sample on the basis of the methylation data, we used the R package glmnet following the approach of Peters et al. (2023) using a binomial elastic net regression with *α* = 0.4 without LOOCV. The sex was encoded as a binary outcome variable (0 = female, 1 = male; Peters et al. 2023). With a predicted probability > 0.5, the sample was male (Peters et al. 2023).

## 3 Results

Following Zoller and Horvath (2024), eight outliers collected between 2001 and 2008 were removed as part of the sample quality assessment (e.g., hierarchical clustering, failed probe fractions, and principal component analysis; Table S2). In addition to these technical outliers, one further animal (KS19‐23Dd) was excluded because of inconsistencies between dental age, pectoral bone age, and number of *corpora albicans*. Accordingly, analyses continued with a total sample size of 75 animals.

### 3.1 Age Prediction

#### 3.1.1 Elastic Net Regression With LOOCV

**FIGURE 1:**
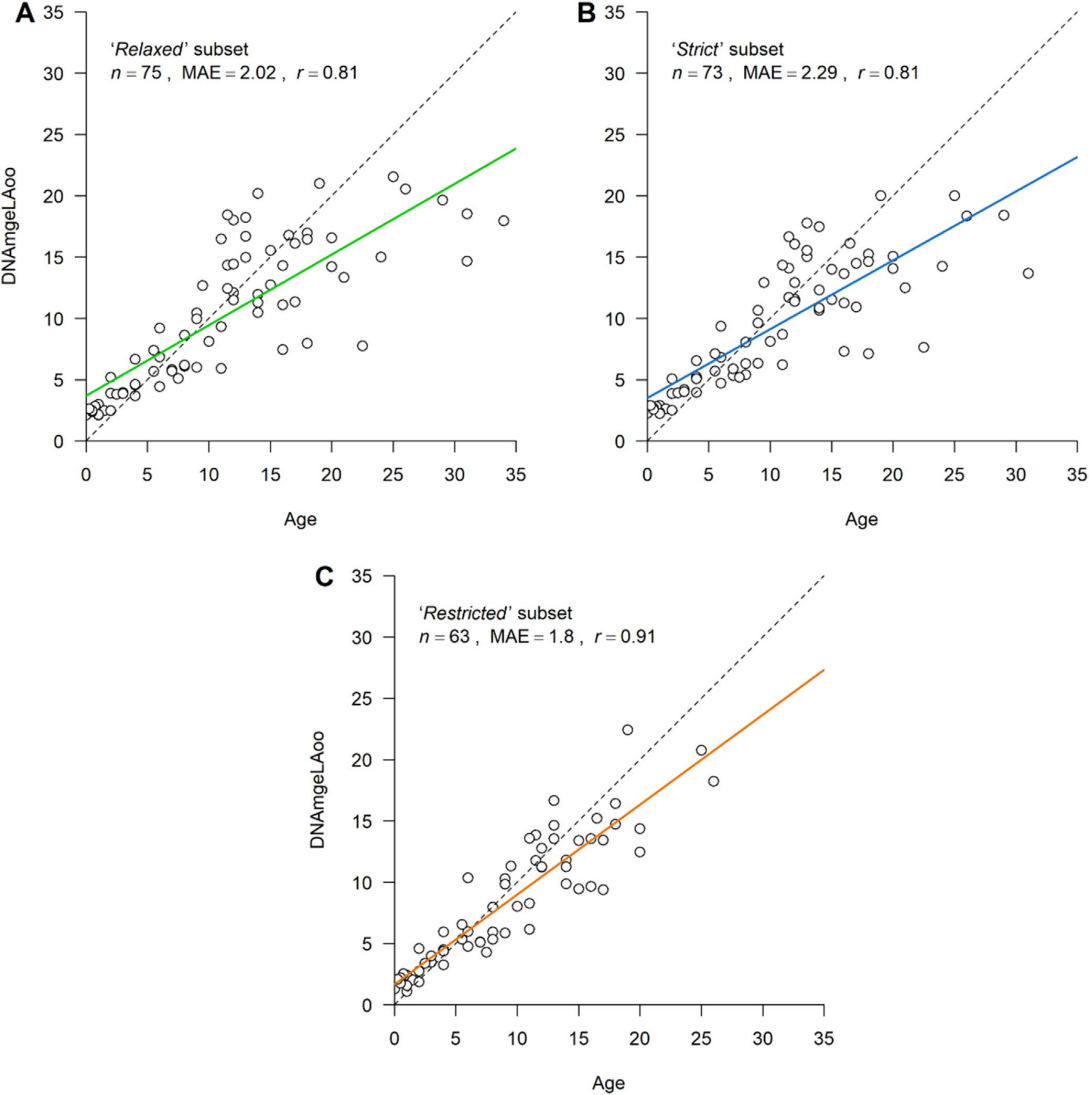
Epigenetic ages (DNAmAgeLOO) estimated using elastic net regression models with Leave‐One‐Out Cross‐Validation (LOOCV) applied to skin samples of common dolphin ( *Delphinus delphis* ) for the (A) *relaxed*, (B) *strict*, and (C) *restricted* subsets. The *relaxed* subset included all individuals, the *strict* subset excluded those with only estimated age ranges or minimum ages, and the *restricted* subset further removed outliers with absolute errors > 6 years. Regression lines are shown in green (*relaxed*), blue (*strict*), or orange (*restricted*), with the dotted diagonal indicating perfect correlation (*y* = *x*). Individual animals are shown as dots. Sample size (*n*), Pearson correlation (*r*), and median absolute error (MAE) are reported for each model.

The elastic net models with LOOCV showed a strong correlation between dental age and predicted age across the different subsets (Figure 1).

For the *relaxed* subset, the elastic net regression retained 38 CpG sites (Table S3) and produced an MAE of 2.02 years, with a correlation of *r* = 0.81 (*p* = 1.39 × 10^−18^) and an *R* ^2^ of 0.66 using a LOOCV. In 12 individuals, the absolute difference between actual and predicted age exceeded 6 years (Table 1). Although predicted age was overestimated in three younger individuals (aged 11.5, 12 and 14 years), most discrepancies (*n* = 9) involved underestimation in older dolphins with a dental age greater than 16 years.

**TABLE 1:** Error metrics for elastic net regression models predicting epigenetic age in common dolphin ( *Delphinus delphis* ), evaluated with Leave‐One‐Out Cross‐Validation (LOOCV) for the *relaxed*, *strict*, and *restricted* subsets.

Metric	Relaxed	Strict	Restricted
Mean absolute error	3.42	3.22	2.21
Median absolute error	2.02	2.29	1.80
N samples with absolute error > 6 years	12	9	4
N samples with absolute error > 10 years	5	4	0
Maximum absolute error	16.31	17.33	7.77

*Note:* The *relaxed* subset included all individuals; the *strict* subset excluded those with estimated age ranges or minimum ages; and the *restricted* subset further removed outliers with errors > 6 years. Shown are the mean and median absolute errors, the number of samples with errors > 6 and 10 years, and the maximum absolute error.

The training set of the *strict* subset retained 24 CpG sites (Table S3) and yielded a MAE of 2.29 years, with a correlation of *r* = 0.81 (*p* = 2.33 × 10^−18^) and an *R* ^2^ of 0.66 using LOOCV. Nine samples, all from individuals older than 16 years, had an absolute error exceeding 6 years, with the predicted ages consistently underestimated in all cases.

For the *restricted* subset, the elastic net model retained 22 CpG sites (Table S3) and demonstrated the best performance, achieving a MAE of 1.80 years, a correlation of *r* = 0.91 (*p* = 1.48 × 10^−24^), and an *R* ^2^ of 0.82 using LOOCV. In this subset, four individuals—each over 16 years of age—had an absolute error exceeding 6 years. Notably, all again were underestimated. A summary of the error metrics of the LOOCV is provided in Table 1.

All elastic nets with LOOCV showed the tendency of overestimating age in younger individuals and underestimating age in older animals (*relaxed*: regression slope: 0.58, regression intercept: 3.69; *strict*: regression slope: 0.56, regression intercept: 3.52; *restricted*: regression slope: 0.73, regression intercept: 1.69). The *restricted* subset has the slope closest to 1, indicating that it exhibits the least amount of bias among the three, performing best in maintaining proportional predictions across ages.

#### 3.1.2 Hybrid Epigenetic Clock

The hybrid model using the *relaxed* subset produced a MAE of 2.61 years (Figure S2) with a correlation of *r* = 0.73 (*p* = 6.16 × 10^−14^). Linear regression of DNA predicted age against dental age yielded a slope of 0.5, an intercept of 4.15, and an *R* ^2^ of 0.54.

#### 3.1.3 Effect of Tissue Decomposition

We found no difference in absolute epigenetic age prediction error across the decomposition condition categories (*p* = 0.746, χ^2^ = 1.23, df = 3), indicating fresh to moderate decomposition change had no overall effect on the accuracy of the epigenetic age estimates.

#### 3.1.4 Effect Storage Duration

Storage duration in the *relaxed* subset ranged from 0 to 23 years (median = 9 years). Spearman correlation indicated no association between storage time and prediction error (*ρ* = 0.10, *p* = 0.38). Similarly, linear regression showed that storage time did not predict error (*β* = 0.029 ± 0.064 SE, *p* = 0.65, *R* ^2^ = 0.003). Residuals from this simple model deviated from normality; however, when chronological age was included as a covariate, residuals conformed to model assumptions (Shapiro–Wilk *p* = 0.10). In this adjusted model, storage time remained non‐significant (*β* = 0.048 ± 0.045 SE, *p* = 0.30), whereas age strongly predicted error (*β* = 0.316 ± 0.036 SE, *p* = 7.3 × 10^−13^, *R* ^2^ = 0.51). Together, these results indicate that storage duration did not confound clock performance in our dataset.

#### 3.1.5 Age Predictions on the Basis of Other Odontocetes Clocks

Age predictions for our samples, generated using both species‐specific and multi‐species odontocete clocks from the Clock Database, are shown in Figure S3, with corresponding error metrics in Table S4.

The multispecies odontocetes clock (Robeck, Fei, Lu, et al. 2021) predicted the age of our samples with an accuracy of *r* = 0.79 for the *relaxed*, *r* = 0.8 for the *strict*, and *r* = 0.85 for the *restricted* subset, and a MAE of 11.8, 11.84, and 12.42, respectively. The use of the multispecies odontocete blood and skin clock resulted in comparable MAE values, but the latter revealed lower age correlations (Table S4). Comparing our results to those of other species‐specific clocks, the Hector's and Māui dolphin ( *Cephalorhynchus hectori hectori* and *C. h. maui*; Hernandez et al. 2023) clock performed best for our samples regarding the MAE between actual and predicted age, with a MAE ranging from 2.71 to 3.01 depending on the subset of samples used (Table S4). Although both multispecies clocks showed the tendency to overestimate the age of our samples, the *Cephalorhynchus* clock consistently underestimated samples above the age of 6 years (see Text S1 and S2).

### 3.2 Sex Prediction

The elastic net model retained 197 CpG sites and achieved 100% accuracy in predicting sex. The probability of males being correctly classified as male ranged from 0.9892 to 0.9953, whereas the probability of females being misclassified as male was very low, ranging from 0.0025 to 0.0078.

## 4 Discussion

Here, we present the first species‐specific epigenetic clock for common dolphins, developed using DNA methylation profiles from skin samples of individuals with estimated dental ages. The elastic net regressions with LOOCV exhibited robust performance across all three models, with the *restricted* subset (MAE = 1.80, *r* = 0.91, *R* ^2^ = 0.82) yielding the highest accuracy with a MAE equivalent to approximately ±5.1% of maximum life expectancy (MLE: 35 years, Murphy et al. 2014). The *relaxed* and *strict* models also performed strongly, with MAEs (2.02 and 2.29 years) equivalent to approx. ±5.8% and ±6.5% of maximum life expectancy, and correlation coefficients of 0.81.

Epigenetic clocks have been shown to estimate chronological age with high accuracy (Jylhävä et al. 2017). However, even when trained on extensive datasets encompassing all age groups, these clocks still exhibit variability among individuals, which could reflect differences in their biological ageing rates (Jylhävä et al. 2017). Variation in ageing rates might be influenced by numerous factors, including reproductive history (Shirazi et al. 2020), early‐life adversities (Colich et al. 2020), nutrition (Fitzgerald et al. 2021; Weindruch et al. 2001), and exposure to environmental contaminants (Liu et al. 2021). In cetaceans, 11 odontocete epigenetic clocks have been developed to date, using a range of sample types including skin, blood, multi‐tissue, and faeces (Table S5). Notably, only three prior studies calibrated their epigenetic clocks using growth layer groups from stranded and bycaught animals, with concerns raised that reliance on tooth age calibration may compromise the robustness of epigenetic clocks (Bors et al. 2021; Hernandez et al. 2023; Mori et al. 2024; Zoller et al. 2025).

Although dental ageing provides robust age estimates for animals younger than 15 years, the reliability of this method decreases in older individuals (Barratclough et al. 2023; Betty et al. 2023; Murphy et al. 2014). Specifically, factors such as tooth wear, the compression of growth layer groups, and the presence of accessory lines can compromise the accuracy of age estimates (Barratclough et al. 2023; Betty et al. 2023; Murphy et al. 2014). Such factors generally result in dental ageing underestimation of older individuals (Barratclough et al. 2023), especially when ageing is restricted to a single tooth, as is typical in studies of animals under human care (Barratclough et al. 2023). To mitigate this, we processed up to three teeth per animal, further selected the least worn and straightest teeth, and assigned minimum age estimates when uncertainty remained because of accessory lines or compressed growth layer groups. Despite these precautions, our epigenetic clock repeatedly underestimated the ages of older individuals (i.e., > 16 years) across all subsets.

The consistent underestimation of older dolphins suggests that discrepancies between dental and epigenetic age assessments unlikely stem from inaccuracies in the dental ageing method itself. Specifically, if dental ageing inaccuracies were the primary source of error, we would expect epigenetic age estimates to exceed dental ages in older individuals—which was not observed. Additionally, contrary to our first hypothesis, overall performance was similar across both the *relaxed* and *strict* subsets. Notably, the *relaxed* model yielded a lower MAE and a regression slope marginally closer to 1, indicating more balanced age predictions across the lifespan. This result suggests that excluding samples with uncertain dental ages did not substantially improve model performance. However, the performance of the *relaxed* model was comparable to most previously published skin‐based odontocete clocks calibrated using growth layer groups, though it fell marginally short of the accuracy reported for clocks developed from known‐age individuals in long‐term observational studies or human care (Text S2). We considered whether this performance gap could be explained by factors relating to tissue origin. Our samples originated from a variety of contexts, including mass and single strandings, bycatch events, and one individual under human care. Although mass strandings are typically unrelated to individual health problems (Cordes 1982), single strandings may involve animals affected by illness, compromised health, or poor nutritional status (Arbelo et al. 2013; Boys 2022). Although poor health has been linked to accelerated epigenetic ageing in other species (Newediuk et al. 2025), this contrasts with the consistent underestimation observed here in older individuals.

We also considered whether post‐mortem decomposition or storage duration could influence methylation patterns. Although post‐mortem changes may potentially affect methylation at specific loci, a comparison of live and deceased human samples showed comparable methylation‐based age predictions (Dias et al. 2020). Consistent with this, we found no significant effect of decomposition condition on epigenetic age prediction error, suggesting that our clock is likely applicable to both live and deceased animals. Similarly, the duration of storage showed no significant association with prediction error, that is, long‐term storage did not affect clock performance.

Beyond biological and technical factors, the composition of the training dataset itself plays a critical role in shaping clock performance. Although our study encompassed the full estimated lifespan of common dolphins, most samples were from individuals aged 0–20 years. Skin samples from older common dolphins remain rare, and the limited and fragmentary representation of older animals may have reduced the accuracy of age estimations in the upper age ranges. When designing epigenetic clocks for wildlife, it is crucial to minimise, where possible, age‐related sampling biases (Newediuk et al. 2025). Specifically, to ensure accurate age predictions, samples should be evenly distributed across age classes, particularly from both young and aged individuals. This prevents poor performance of clocks because of narrow or skewed age distributions (Newediuk et al. 2025). However, the heterogeneous age distribution of stranded and bycaught individuals relies upon extensive tissue archives, which capture the full age spectrum of the species.

In our study, regression analyses revealed slopes smaller than 1 for all three models, indicating a systematic overestimation of age in younger individuals and underestimation in older individuals. This pattern is consistent across multiple species (Beal et al. 2019; Bors et al. 2021; Peters et al. 2023; Polanowski et al. 2014). Among our models, the *restricted* model showed the slope closest to 1, suggesting the most balanced age predictions across the lifespan. The phenomenon of “regression to the mean” poses a well‐recognised challenge for epigenetic clock models, particularly when predicting ages beyond the range of the training data. This statistical effect causes extreme values to shift toward the population mean, leading to underestimation of older individuals and overestimation of younger ones when these age groups are underrepresented in the training set. In human studies, this has prompted efforts to develop specialised clocks for centenarians (Dec et al. 2023). Indeed, this concept and its implications for research design have been widely discussed (Campbell and Kenny 1999). Accordingly, when interpreting epigenetic age estimates for individuals at the extremes of the age spectrum, it is important to account for the influence of regression to the mean and its bias toward the average age of the training cohort.

Regression to the mean, because of the sparse representation of older individuals, is one explanation for the underestimation of age at higher values. Another plausible factor is the biological deceleration of DNA methylation change in later life. For example, human epigenetic clocks have been shown to progress rapidly during development and then decelerate, leading to a systematic underestimation of age in older individuals (Horvath 2013; Kuzub et al. 2022; Marioni et al. 2019). If odontocetes exhibit a comparable biological deceleration, it would systematically bias clock estimates downward in older dolphins, regardless of sample distribution. This has also been hypothesised in bottlenose dolphins, where the use of hybrid models has been suggested as a strategy to address both uneven age distributions and life stage–dependent variation in methylation change (Barratclough et al. 2024).

Hybrid models aim to improve predictions across the lifespan by adjusting for shifts in methylation patterns after physical maturity. Barratclough et al. (2024) applied this strategy in bottlenose dolphins ( *Tursiops truncatus* ), combining a random forest classifier with two elastic net regression models to improve age prediction for older individuals. However, applying a similar approach to common dolphins yielded a poorer fit than elastic net regression alone, likely because of the small number of physically mature individuals in our dataset (*n* = 14), limiting the classifier's and mature‐age model's effectiveness. Future approaches, such as learning curves (Perlich et al. 2003) or bias–variance decomposition (Domingos 2000), could be used to disentangle whether underperformance reflects model bias or limited sample size when sufficient sample sizes permit.

Despite limitations identified, our results highlight the promise of epigenetic clocks on the basis of dental ageing in addressing long‐standing challenges in age estimation, particularly for species where invasive sampling or long‐term observational data are not feasible. Beyond age estimation, our clock also accurately predicts sex on the basis of methylation patterns at CpG sites, achieving 100% accuracy, a finding consistent with previous studies (Bors et al. 2021; Peters et al. 2023; Robeck, Fei, Haghani, et al. 2021). Tools that enable accurate age and sex estimation are becoming increasingly critical, given the significant anthropogenic threats facing common dolphins globally (Abraham et al. 2021; Bilgmann et al. 2014; Fernández‐Contreras et al. 2010; Hamer et al. 2007; Mannocci et al. 2012; Murphy et al. 2013; Mussi et al. 2021; Peltier et al. 2016, 2021; Piroddi et al. 2011; Tulloch et al. 2020; Vella et al. 2021), where several populations are in decline (Bearzi et al. 2008; Murphy et al. 2013; Piroddi et al. 2011). Understanding life‐history parameters is critical for conservation management (Betty et al. 2019; Heydenrych et al. 2021; Manlik et al. 2022). For example, accurate age estimates underpin population viability analyses and are essential to quantify population‐level impacts such as fisheries bycatch (Manlik et al. 2016; Palmer et al. 2022; Verborgh et al. 2021). Since post‐mortem age assessment may bias survivorship estimates (Betty et al. 2023) used to evaluate fisheries impacts, our epigenetic clock provides a critical advance by enabling age estimation from living animals.

The ability to age live individuals strengthens conservation efforts globally. Life‐history traits are comparable among common dolphin populations worldwide (Danil and Chivers 2007; Murphy and Rogan 2006; Palmer et al. 2022, 2023; Westgate and Read 2007), and species‐specific epigenetic clocks have been shown to accurately predict age in subspecies and closely related sister taxa (Barratclough et al. 2024, 2025; Peters et al. 2023; Robeck, Fei, Lu, et al. 2021). Our epigenetic clock has broad applicability for this cosmopolitan oceanic species and thus may further facilitate comparisons of epigenetic age acceleration across populations experiencing varying environmental pressures (Newediuk et al. 2025). By enabling reliable age and sex estimation, this tool can enhance conservation strategies and improve our understanding of population dynamics, both in Aotearoa, New Zealand, and throughout the species' range. Finally, with concerns of decomposition and tooth ageing effects on clock calibration addressed here, future methylated clocks should be considered for stranded and bycaught dolphin populations, especially those underpinned by longitudinal tissue archives that allow teeth estimates to be further cross‐validated with wider life‐history data.

## Author Contributions

**Eva‐Maria F. Hanninger:** conceptualization (lead), data curation (lead), formal analysis (lead), funding acquisition (lead), investigation (lead), methodology (lead), resources (lead), software (lead), validation (lead), visualization (lead), writing – original draft (lead), writing – review and editing (lead). **Katharina J. Peters:** conceptualization (equal), formal analysis (supporting), investigation (supporting), methodology (supporting), supervision (equal), writing – original draft (supporting), writing – review and editing (supporting). **Livia Gerber:** conceptualization (supporting), formal analysis (supporting), methodology (supporting), writing – original draft (supporting), writing – review and editing (supporting). **Ashley Barratclough:** formal analysis (supporting), methodology (supporting), writing – original draft (supporting), writing – review and editing (supporting). **Emma L. Betty:** methodology (equal), writing – original draft (supporting), writing – review and editing (supporting). **Emily I. Palmer:** methodology (equal), writing – original draft (supporting), writing – review and editing (supporting). **Steve Horvath:** writing – original draft (supporting), writing – review and editing (supporting). **Karen A. Stockin:** conceptualization (equal), data curation (equal), funding acquisition (equal), methodology (supporting), resources (lead), supervision (lead), writing – original draft (supporting).

## Conflicts of Interest

The Regents of the University of California are the sole owners of patents and patent applications directed at epigenetic biomarkers for which Steve Horvath is a named inventor; S.H. is a founder and paid consultant of the non‐profit Epigenetic Clock Development Foundation that licenses these patents. S.H. is a Principal Investigator at Altos Labs, Cambridge Institute of Science, a biomedical company that works on rejuvenation.

## Supporting information


**Appendix S1:** ece372424‐sup‐0001‐AppendixS1.docx.

## Data Availability

All data and R code supporting this study are accessible at https://github.com/Ehanninger/Epigenetic‐clock‐Delphinus‐delphis. The dataset will further be accessible through the Mammalian Methylation Consortium's data release. The mammalian methylation array used in this research is available from the non‐profit Epigenetic Clock Development Foundation.

## Endnotes

^1^

https://genomics.senescence.info/species/index.html.

## References

[ece372424-bib-0001] Abraham, E. R. , L. Tremblay‐Boyer , and K. Berkenbusch . 2021. “Estimated Captures of New Zealand Fur Seal, Common Dolphin, and Turtles in New Zealand Commercial Fisheries, to 2017–18.” http://www.mpi.govt.nz/news‐and‐resources/publications.

[ece372424-bib-0002] Arbelo, M. , A. Espinosa de los Monteros , P. Herráez , et al. 2013. “Pathology and Causes of Death of Stranded Cetaceans in the Canary Islands (1999‐2005).” Diseases of Aquatic Organisms 103, no. 2: 87–99. 10.3354/dao02558. 23548359

[ece372424-bib-0003] Arneson, A. , A. Haghani , M. J. Thompson , et al. 2022. “A Mammalian Methylation Array for Profiling Methylation Levels at Conserved Sequences.” Nature Communications 13, no. 1: 783. 10.1038/s41467-022-28355-z. PMC8831611 35145108

[ece372424-bib-0004] Barratclough, A. , R. C. Genoves , L. Schwacke , et al. 2025. “Application of Epigenetics to Understand the Demography of the Lahille's Bottlenose Dolphin *Tursiops truncatus gephyreus* .” Endangered Species Research 58: 137–146. 10.3354/esr01441.

[ece372424-bib-0005] Barratclough, A. , W. E. McFee , M. Stolen , et al. 2023. “How to Estimate Age of Old Bottlenose Dolphins (*Tursiops truncatus*); by Tooth or Pectoral Flipper?” Frontiers in Marine Science 10: 1135521. 10.3389/fmars.2023.1135521.

[ece372424-bib-0006] Barratclough, A. , R. Sanz‐Requena , L. Marti‐Bonmati , T. L. Schmitt , E. Jensen , and D. García‐Párraga . 2019. “Radiographic Assessment of Pectoral Flipper Bone Maturation in Bottlenose Dolphins ( *Tursiops truncatus* ), as a Novel Technique to Accurately Estimate Chronological Age.” PLoS One 14, no. 9: e0222722. 10.1371/journal.pone.0222722. 31557197 PMC6762177

[ece372424-bib-0007] Barratclough, A. , C. R. Smith , F. M. Gomez , et al. 2021. “Accurate Epigenetic Aging in Bottlenose Dolphins ( *Tursiops truncatus* ), an Essential Step in the Conservation of At‐Risk Dolphins.” Journal of Zoological and Botanical Gardens 2, no. 3: 416–420. 10.3390/jzbg2030030.

[ece372424-bib-0008] Barratclough, A. , R. Takeshita , L. Thomas , et al. 2024. “Estimating Age and Investigating Epigenetic Changes Related to Health Across Multiple Bottlenose Dolphin Populations.” Biological Conservation 293: 110570. 10.1016/j.biocon.2024.110570.

[ece372424-bib-0009] Barratclough, A. , R. S. Wells , L. H. Schwacke , et al. 2019. “Health Assessments of Common Bottlenose Dolphins ( *Tursiops truncatus* ): Past, Present, and Potential Conservation Applications.” Frontiers in Veterinary Science 6: 444. 10.3389/fvets.2019.00444. 31921905 PMC6923228

[ece372424-bib-0010] Beal, A. P. , J. J. Kiszka , R. S. Wells , and J. M. Eirin‐Lopez . 2019. “The Bottlenose Dolphin Epigenetic Aging Tool (BEAT): A Molecular Age Estimation Tool for Small Cetaceans.” Frontiers in Marine Science 6: 561. 10.3389/fmars.2019.00561.

[ece372424-bib-0011] Bearzi, G. , S. Agazzi , J. Gonzalvo , et al. 2008. “Overfishing and the Disappearance of Short‐Beaked Common Dolphins From Western Greece.” Endangered Species Research 5: 1–12. 10.3354/esr00103.

[ece372424-bib-0013] Betty, E. L. , K. A. Stockin , B. Hinton , et al. 2022. “Age, Growth, and Sexual Dimorphism of the Southern Hemisphere Long‐Finned Pilot Whale (*Globicephala melas edwardii*).” Journal of Mammalogy 103, no. 3: 560–575. 10.1093/jmammal/gyab165. 35707679 PMC9189691

[ece372424-bib-0012] Betty, E. L. , K. A. Stockin , B. Hinton , B. A. Bollard , M. B. Orams , and S. Murphy . 2023. “Age‐ and Sex‐Specific Survivorship of the Southern Hemisphere Long‐Finned Pilot Whale ( *Globicephala melas edwardii* ).” Journal of Mammalogy 104, no. 1: 39–48. 10.1093/jmammal/gyac085. 36818685 PMC9936501

[ece372424-bib-0014] Betty, E. L. , K. A. Stockin , A. N. H. Smith , B. Bollard , M. B. Orams , and S. Murphy . 2019. “Sexual Maturation in Male Long‐Finned Pilot Whales ( *Globicephala melas edwardii* ): Defining Indicators of Sexual Maturity.” Journal of Mammalogy 100, no. 4: 1387–1402. 10.1093/jmammal/gyz086.

[ece372424-bib-0015] Bilgmann, K. , G. J. Parra , N. Zanardo , L. B. Beheregaray , and L. M. Möller . 2014. “Multiple Management Units of Short‐Beaked Common Dolphins Subject to Fisheries Bycatch Off Southern and Southeastern Australia.” Marine Ecology Progress Series 500: 265–279. 10.3354/meps10649.

[ece372424-bib-0016] Bocklandt, S. , W. Lin , M. E. Sehl , et al. 2011. “Epigenetic Predictor of Age.” PLoS One 6, no. 6: e14821. 10.1371/journal.pone.0014821. 21731603 PMC3120753

[ece372424-bib-0017] Bors, E. K. , C. S. Baker , P. R. Wade , et al. 2021. “An Epigenetic Clock to Estimate the Age of Living Beluga Whales.” Evolutionary Applications 14, no. 5: 1263–1273. 10.1111/eva.13195. 34025766 PMC8127720

[ece372424-bib-0018] Boys, R. M. 2022. Applying Welfare Science to Cetacean Strandings Doctoral Thesis. Massey University.

[ece372424-bib-0019] Campbell, D. T. , and D. A. Kenny . 1999. A Primer on Regression Artifacts. Guilford Press.

[ece372424-bib-0020] Chivers, S. J. 2018. “Cetacean Life History.” In Encyclopedia of Marine Mammals, 186–189. Elsevier. 10.1016/B978-0-12-804327-1.00089-3.

[ece372424-bib-0021] Colich, N. L. , M. L. Rosen , E. S. Williams , and K. A. McLaughlin . 2020. “Biological Aging in Childhood and Adolescence Following Experiences of Threat and Deprivation: A Systematic Review and Meta‐Analysis.” Psychological Bulletin 146, no. 9: 721–764. 10.1037/bul0000270. 32744840 PMC7484378

[ece372424-bib-0022] Connor, R. C. , and M. Krützen . 2015. “Male Dolphin Alliances in Shark Bay: Changing Perspectives in a 30‐Year Study.” Animal Behaviour 103: 223–235. 10.1016/j.anbehav.2015.02.019.

[ece372424-bib-0023] Cordes, D. O. 1982. “The Causes of Whale Strandings.” New Zealand Veterinary Journal 30, no. 3: 21–24. 10.1080/00480169.1982.34865. 16030851

[ece372424-bib-0024] Danil, K. , and S. J. Chivers . 2007. “Growth and Reproduction of Female Short‐Beaked Common Dolphins, *Delphinus delphis* , in the Eastern Tropical Pacific.” Canadian Journal of Zoology 85, no. 1: 108–121. 10.1139/z06-188.

[ece372424-bib-0025] De Paoli‐Iseppi, R. , B. E. Deagle , C. R. McMahon , M. A. Hindell , J. L. Dickinson , and S. N. Jarman . 2017. “Measuring Animal Age With DNA Methylation: From Humans to Wild Animals.” Frontiers in Genetics 8: 106. 10.3389/fgene.2017.00106. 28878806 PMC5572392

[ece372424-bib-0026] Dec, E. , J. Clement , K. Cheng , et al. 2023. “Centenarian Clocks: Epigenetic Clocks for Validating Claims of Exceptional Longevity.” GeroScience 45, no. 3: 1817–1835. 10.1007/s11357-023-00731-7. 36964402 PMC10400760

[ece372424-bib-0027] Desjardins, P. , and D. Conklin . 2010. “NanoDrop Microvolume Quantitation of Nucleic Acids.” Journal of Visualized Experiments 1. 10.3791/2565. PMC3346308 21189466

[ece372424-bib-0028] Dias, H. C. , C. Cordeiro , J. Pereira , et al. 2020. “DNA Methylation Age Estimation in Blood Samples of Living and Deceased Individuals Using a Multiplex SNaPshot Assay.” Forensic Science International 311: 110267. 10.1016/j.forsciint.2020.110267. 32325350

[ece372424-bib-0029] Domingos, P. 2000. “A Unified Bias‐Variance Decomposition.” In Proceedings of 17th International Conference on Machine Learning, 231–238. Morgan Kaufmann Publishers Inc.

[ece372424-bib-0030] Evans, K. , M. A. Hindell , K. Robertson , C. Lockyer , and D. Rice . 2002. “Factors Affecting the Precision of Age Determination of Sperm Whales (*Physeter macrocephalus*).” Journal of Cetacean Research and Management 4, no. 2: 193–201.

[ece372424-bib-0031] Fernández‐Contreras, M. M. , L. Cardona , C. H. Lockyer , and A. Aguilar . 2010. “Incidental Bycatch of Short‐Beaked Common Dolphins ( *Delphinus delphis* ) by Pairtrawlers off Northwestern Spain.” ICES Journal of Marine Science 67, no. 8: 1732–1738. 10.1093/icesjms/fsq077.

[ece372424-bib-0032] Fitzgerald, K. N. , R. Hodges , D. Hanes , et al. 2021. “Potential Reversal of Epigenetic Age Using a Diet and Lifestyle Intervention: A Pilot Randomized Clinical Trial.” Aging 13, no. 7: 9419–9432. 10.18632/aging.202913. 33844651 PMC8064200

[ece372424-bib-0033] Friedman, J. , T. Hastie , and R. Tibshirani . 2010. “Regularization Paths for Generalized Linear Models via Coordinate Descent.” Journal of Statistical Software 33, no. 1: 1–22. https://pubmed.ncbi.nlm.nih.gov/20808728/. 20808728 PMC2929880

[ece372424-bib-0034] Goren, A. D. , P. F. Brodie , S. Spotte , et al. 1987. “Growth Layer Groups (GLGs) in the Teeth of an Adult Beluga Whale (*Delphinapterus leucas*) of Known Age: Evidence for Two Annual Layers.” Marine Mammal Science 3, no. 1: 14–21. 10.1111/j.1748-7692.1987.tb00148.x.

[ece372424-bib-0035] Guevara, E. E. , and R. R. Lawler . 2018. “Epigenetic Clocks.” Evolutionary Anthropology 27, no. 6: 256–260. 10.1002/evan.21745. 30383910

[ece372424-bib-0036] Haghani, A. , C. Z. Li , T. R. Robeck , et al. 2023. “DNA Methylation Networks Underlying Mammalian Traits.” Science 381, no. 6658: eabq5693. 10.1126/science.abq5693. 37561875 PMC11180965

[ece372424-bib-0037] Hamer, D. J. , T. M. Ward , S. D. Goldsworthy , R. Mcgarvey , and P. J. Rogers . 2007. “Measurement, Management and Mitigation of Operational Interactions Between Common Dolphins (*Delphinus delphis*) and the South Australian Sardine Fishery.” Report to PIRSA Fisheries. 10.1016/j.biocon.2008.08.024.

[ece372424-bib-0038] Hernandez, K. M. , K. B. O'Neill , E. K. Bors , et al. 2023. “Using Epigenetic Clocks to Investigate Changes in the Age Structure of Critically Endangered Māui Dolphins.” Ecology and Evolution 13, no. 10: e10562. 10.1002/ece3.10562. 37780090 PMC10534197

[ece372424-bib-0039] Heydenrych, M. J. , B. J. Saunders , M. Bunce , and S. N. Jarman . 2021. “Epigenetic Measurement of Key Vertebrate Population Biology Parameters.” Frontiers in Ecology and Evolution 9: 617376. 10.3389/fevo.2021.617376.

[ece372424-bib-0040] Horvath, S. 2013. “DNA Methylation Age of Human Tissues and Cell Types.” Genome Biology 14, no. 10: 3156. 10.1186/gb-2013-14-10-r115. PMC4015143 24138928

[ece372424-bib-0041] Horvath, S. , and K. Raj . 2018. “DNA Methylation‐Based Biomarkers and the Epigenetic Clock Theory of Ageing.” Nature Reviews Genetics 19, no. 6: 371–384. 10.1038/s41576-018-0004-3. 29643443

[ece372424-bib-0042] IJsseldijk, L. L. , A. C. Brownlow , and S. Mazzariol . 2019. “Best Practice on Cetacean Post‐Mortem Investigation and Tissue Sampling.” https://osf.io/zh4ra/.

[ece372424-bib-0043] Issa, J. P. 2014. “Aging and Epigenetic Drift: A Vicious Cycle.” Journal of Clinical Investigation 124, no. 1: 24–29. 10.1172/JCI69735. 24382386 PMC3871228

[ece372424-bib-0044] Jylhävä, J. , N. L. Pedersen , and S. Hägg . 2017. “Biological Age Predictors.” eBioMedicine 21: 29–36. 10.1016/j.ebiom.2017.03.046. 28396265 PMC5514388

[ece372424-bib-0045] Krzyszczyk, E. , and J. Mann . 2012. “Why Become Speckled? Ontogeny and Function of Speckling in Shark Bay Bottlenose Dolphins (*Tursiops* sp.).” Marine Mammal Science 28, no. 2: 295–307. 10.1111/j.1748-7692.2011.00483.x.

[ece372424-bib-0046] Kuzub, N. , V. Smialkovska , V. Momot , V. Moseiko , O. Lushchak , and A. Koliada . 2022. “Evaluation of Epigenetic Age Based on DNA Methylation Analysis of Several CpG Sites in Ukrainian Population.” Frontiers in Genetics 12: 772298. 10.3389/fgene.2021.772298. 35069680 PMC8770732

[ece372424-bib-0047] Liu, Y. , J. Wang , Z. Huang , et al. 2021. “Environmental Pollutants Exposure: A Potential Contributor for Aging and Age‐Related Diseases.” Environmental Toxicology and Pharmacology 83: 103575. 10.1016/j.etap.2020.103575. 33385577

[ece372424-bib-0048] Lockyer, C. H. 1993. “A Report on Patterns of Deposition of Dentine and Cement in Teeth of Pilot Whales, Genus *Globicephala* .” Reports of the International Whaling Commission, Special Issue 14: 137–161.

[ece372424-bib-0049] Lockyer, C. H. 1995. “A Review of Factors Involved in Zonation in Odontocete Teeth, and an Investigation of the Likely Impact of Environmental Factors and Major Life Events on Harbour Porpoise Tooth Structure.” Oceanographic Literature Review 10: 1042.

[ece372424-bib-0050] Lu, A. T. , Z. Fei , A. Haghani , et al. 2023. “Universal DNA Methylation Age Across Mammalian Tissues.” Nature Aging 3: 1144–1166. 10.1038/s43587-023-00462-6. 37563227 PMC10501909

[ece372424-bib-0051] Maas, M. C. 2009. “Bones and Teeth, Histology of.” In Encyclopedia of Marine Mammals, 124–129. Elsevier. 10.1016/B978-0-12-373553-9.00034-1.

[ece372424-bib-0052] Manlik, O. , R. C. Lacy , W. B. Sherwin , H. Finn , N. R. Loneragan , and S. J. Allen . 2022. “A Stochastic Model for Estimating Sustainable Limits to Wildlife Mortality in a Changing World.” Conservation Biology 36, no. 4: e13897. 10.1111/cobi.13897. 35122329 PMC9542519

[ece372424-bib-0053] Manlik, O. , J. A. Mcdonald , J. Mann , et al. 2016. “The Relative Importance of Reproduction and Survival for the Conservation of Two Dolphin Populations.” Ecology and Evolution 6, no. 11: 3496–3512. 10.1002/ece3.2130. 28725349 PMC5513288

[ece372424-bib-0054] Mannocci, L. , W. Dabin , E. Augeraud‐Véron , J. F. Dupuy , C. Barbraud , and V. Ridoux . 2012. “Assessing the Impact of Bycatch on Dolphin Populations: The Case of the Common Dolphin in the Eastern North Atlantic.” PLoS One 7, no. 2: e32615. 10.1371/journal.pone.0032615. 22393423 PMC3290591

[ece372424-bib-0055] Marioni, R. E. , M. Suderman , B. H. Chen , et al. 2019. “Tracking the Epigenetic Clock Across the Human Life Course: A Meta‐Analysis of Longitudinal Cohort Data.” Journals of Gerontology: Series A 74, no. 1: 57–61. 10.1093/gerona/gly060. PMC6298183 29718110

[ece372424-bib-0056] Mayne, B. , O. Berry , and S. Jarman . 2021. “Optimal Sample Size for Calibrating DNA Methylation Age Estimators.” Molecular Ecology Resources 21, no. 7: 2316–2323. 10.1111/1755-0998.13437. 34053192 PMC8518423

[ece372424-bib-0057] Mayne, B. , O. Berry , and S. Jarman . 2023. “Calibrating Epigenetic Clocks With Training Data Error.” Evolutionary Applications 16, no. 8: 1496–1502. 10.1111/eva.13582. 37622096 PMC10445086

[ece372424-bib-0058] Mishra, S. , U. Sarkar , S. Taraphder , et al. 2017. “Principal Component Analysis.” International Journal of Livestock Research 1: 1.

[ece372424-bib-0059] Moore, J. E. , and A. J. Read . 2008. “A Bayesian Uncertainty Analysis of Cetacean Demography and Bycatch Mortality Using Age‐At‐Death Data.” Ecological Applications 18, no. 8: 1914–1931. 10.1890/07-0862.1. 19263888

[ece372424-bib-0060] Mori, M. , H. Maeda , A. Tanabe , et al. 2024. “Estimation of Chronological Age of Risso's Dolphin (*Grampus griseus*) Based on DNA Methylation Frequency.” Frontiers in Marine Science 11: 1391674. 10.3389/fmars.2024.1391674.

[ece372424-bib-0061] Murphy, S. , R. J. Law , R. Deaville , et al. 2018. “Organochlorine Contaminants and Reproductive Implication in Cetaceans.” In Marine Mammal Ecotoxicology, 3–38. Elsevier. 10.1016/B978-0-12-812144-3.00001-2.

[ece372424-bib-0062] Murphy, S. , M. Perrott , J. McVee , F. L. Read , and K. A. Stockin . 2014. “Deposition of Growth Layer Groups in Dentine Tissue of Captive Common Dolphins *Delphinus delphis* .” NAMMCO Scientific Publications 8. 10.7557/3.3017.

[ece372424-bib-0063] Murphy, S. , E. H. Pinn , and P. D. Jepson . 2013. “The Short‐Beaked Common Dolphin (*Delphinus delphis*) in the North‐East Atlantic: Distribution, Ecology, Management and Conservation Status.” Oceanography and Marine Biology: An Annual Review 51: 193–280.

[ece372424-bib-0064] Murphy, S. , and E. Rogan . 2006. “External Morphology of the Short‐Beaked Common Dolphin, *Delphinus delphis*: Growth, Allometric Relationships and Sexual Dimorphism.” Acta Zoologica 87, no. 4: 315–329. 10.1111/j.1463-6395.2006.00245.x.

[ece372424-bib-0065] Mussi, B. , C. Vivaldi , A. Zucchini , A. Miragliuolo , and D. S. Pace . 2021. “The Decline of Short‐Beaked Common Dolphin ( *Delphinus delphis* ) in the Waters off the Island of Ischia (Gulf of Naples, Italy).” Aquatic Conservation: Marine and Freshwater Ecosystems 31, no. S1: 87–100. 10.1002/aqc.3061.

[ece372424-bib-0066] Newediuk, L. , E. S. Richardson , A. M. Bohart , A. Roberto‐Charron , C. J. Garroway , and M. J. Jones . 2025. “Designing Epigenetic Clocks for Wildlife Research.” Molecular Ecology Resources 25: e14120. 10.1111/1755-0998.14120. 40326643 PMC12415925

[ece372424-bib-0067] Palmer, E. I. 2023. Life History of Common Dolphins (*Delphinus delphis*) in Aotearoa New Zealand. Doctoral Thesis. Massey University.

[ece372424-bib-0068] Palmer, E. I. , E. L. Betty , S. Murphy , M. R. Perrott , A. N. H. Smith , and K. A. Stockin . 2022. “Reproductive Biology of Female Common Dolphins ( *Delphinus delphis* ) in New Zealand Waters.” Marine Biology 169, no. 12: 158. 10.1007/s00227-022-04139-3. 36466079 PMC9705467

[ece372424-bib-0069] Palmer, E. I. , E. L. Betty , S. Murphy , M. R. Perrott , A. N. H. Smith , and K. A. Stockin . 2023. “Reproductive Biology of Male Common Dolphins ( *Delphinus delphis* ) in New Zealand Waters.” Marine Biology 170, no. 12: 153. 10.1007/s00227-023-04266-5. 37811127 PMC10558376

[ece372424-bib-0070] Parsons, K. M. , A. Haghani , J. A. Zoller , et al. 2023. “DNA Methylation‐Based Biomarkers for Ageing Long‐Lived Cetaceans.” Molecular Ecology Resources 23, no. 6: 1241–1256. 10.1111/1755-0998.13791. 36994812

[ece372424-bib-0071] Peltier, H. , M. Authier , F. Caurant , et al. 2021. “In the Wrong Place at the Wrong Time: Identifying Spatiotemporal Co‐occurrence of Bycaught Common Dolphins and Fisheries in the Bay of Biscay (NE Atlantic) From 2010 to 2019.” Frontiers in Marine Science 8: 617342. 10.3389/fmars.2021.617342.

[ece372424-bib-0072] Peltier, H. , M. Authier , R. Deaville , et al. 2016. “Small Cetacean Bycatch as Estimated From Stranding Schemes: The Common Dolphin Case in the Northeast Atlantic.” Environmental Science & Policy 63: 7–18. 10.1016/j.envsci.2016.05.004.

[ece372424-bib-0073] Perlich, C. , F. Provost , and J. S. Simonoff . 2003. “Tree Induction vs. Logistic Regression: A Learning‐Curve Analysis.” Journal of Machine Learning Research 4: 211–255. https://dl.acm.org/doi/10.1162/153244304322972694.

[ece372424-bib-0074] Perrin, W. F. 2009. “Common Dolphins.” In Encyclopedia of Marine Mammals, 255–259. Elsevier. 10.1016/B978-0-12-373553-9.00063-8.

[ece372424-bib-0075] Perrin, W. F. , and A. C. J. Myrick . 1980. “Report of the Workshop. Age Determination of Toothed Whales and Sirenians.” Reports of the International Whaling Commission 3: 1–50.

[ece372424-bib-0076] Peters, K. J. , L. Gerber , L. Scheu , et al. 2023. “An Epigenetic DNA Methylation Clock for Age Estimates in Indo‐Pacific Bottlenose Dolphins ( *Tursiops aduncus* ).” Evolutionary Applications 16, no. 1: 126–133. 10.1111/eva.13516. 36699128 PMC9850008

[ece372424-bib-0077] Piroddi, C. , G. Bearzi , J. Gonzalvo , and V. Christensen . 2011. “From Common to Rare: The Case of the Mediterranean Common Dolphin.” Biological Conservation 144, no. 10: 2490–2498. 10.1016/j.biocon.2011.07.003.

[ece372424-bib-0078] Polanowski, A. M. , J. Robbins , D. Chandler , and S. N. Jarman . 2014. “Epigenetic Estimation of Age in Humpback Whales.” Molecular Ecology Resources 14, no. 5: 976–987. 10.1111/1755-0998.12247. 24606053 PMC4314680

[ece372424-bib-0079] Poulsen, P. , M. Esteller , A. Vaag , and M. F. Fraga . 2007. “The Epigenetic Basis of Twin Discordance in Age‐Related Diseases.” Pediatric Research 61, no. 5 Pt 2: 38R–42R. 10.1203/pdr.0b013e31803c7b98. 17413848

[ece372424-bib-0080] Read, F. L. , A. A. Hohn , and C. H. Lockyer . 2018. “A Review of Age Estimation Methods in Marine Mammals With Special Reference to Monodontids.” NAMMCO Scientific Publications 10. 10.7557/3.4474.

[ece372424-bib-0081] Robeck, T. R. , Z. Fei , A. Haghani , et al. 2021. “Multi‐Tissue Methylation Clocks for Age and Sex Estimation in the Common Bottlenose Dolphin.” Frontiers in Marine Science 8: 713373. 10.3389/fmars.2021.713373.

[ece372424-bib-0082] Robeck, T. R. , Z. Fei , A. T. Lu , et al. 2021. “Multi‐Species and Multi‐Tissue Methylation Clocks for Age Estimation in Toothed Whales and Dolphins.” Communications Biology 4, no. 1: 642. 10.1038/s42003-021-02179-x. 34059764 PMC8167141

[ece372424-bib-0083] Sen, P. , P. P. Shah , R. Nativio , and S. L. Berger . 2016. “Epigenetic Mechanisms of Longevity and Aging.” Cell 166, no. 4: 822–839. 10.1016/j.cell.2016.07.050. 27518561 PMC5821249

[ece372424-bib-0084] Shirazi, T. N. , W. J. Hastings , A. Y. Rosinger , and C. P. Ryan . 2020. “Parity Predicts Biological Age Acceleration in Post‐Menopausal, but Not Pre‐Menopausal, Women.” Scientific Reports 10, no. 1: 20522. 10.1038/s41598-020-77082-2. 33239686 PMC7689483

[ece372424-bib-0085] Simpson, D. J. , and T. Chandra . 2021. “Epigenetic Age Prediction.” Aging Cell 20, no. 9: e13452. 10.1111/acel.13452. 34415665 PMC8441394

[ece372424-bib-0086] Stockin, K. A. , P. J. Duignan , W. D. Roe , L. Meynier , M. Alley , and T. Fettermann . 2009. “Causes of Mortality in Stranded Common Dolphin (*Delphinus sp*.) From New Zealand Waters Between 1998 and 2008.” Pacific Conservation Biology 15, no. 3: 217. 10.1071/PC090217.

[ece372424-bib-0087] Stockin, K. A. , R. J. Law , W. D. Roe , et al. 2010. “PCBs and Organochlorine Pesticides in Hector's ( *Cephalorhynchus hectori hectori* ) and Maui's ( *Cephalorhynchus hectori maui* ) Dolphins.” Marine Pollution Bulletin 60, no. 6: 834–842. 10.1016/j.marpolbul.2010.01.009. 20226477

[ece372424-bib-0088] Teschendorff, A. E. , and S. Horvath . 2025. “Epigenetic Ageing Clocks: Statistical Methods and Emerging Computational Challenges.” Nature Reviews Genetics 26, no. 5: 350–368. 10.1038/s41576-024-00807-w. 39806006

[ece372424-bib-0089] Tulloch, V. , V. Pirotta , A. Grech , et al. 2020. “Long‐Term Trends and a Risk Analysis of Cetacean Entanglements and Bycatch in Fisheries Gear in Australian Waters.” Biodiversity and Conservation 29, no. 1: 251–282. 10.1007/s10531-019-01881-x.

[ece372424-bib-0090] Vella, A. , S. Murphy , J. Giménez , et al. 2021. “The Conservation of the Endangered Mediterranean Common Dolphin ( *Delphinus delphis* ): Current Knowledge and Research Priorities.” Aquatic Conservation: Marine and Freshwater Ecosystems 31, no. S1: 110–136. 10.1002/aqc.3538.

[ece372424-bib-0091] Verborgh, P. , P. Gauffier , R. Esteban , and R. de Stephanis . 2021. “Demographic Parameters of a Free‐Ranging Deep‐Diving Cetacean, the Long‐Finned Pilot Whale.” Marine Mammal Science 37, no. 2: 463–481. 10.1111/mms.12752.

[ece372424-bib-0092] Waugh, D. A. , R. S. Suydam , J. D. Ortiz , and J. G. M. Thewissen . 2018. “Validation of Growth Layer Group (GLG) Depositional Rate Using Daily Incremental Growth Lines in the Dentin of Beluga ( *Delphinapterus leucas* (Pallas, 1776)) Teeth.” PLoS One 13, no. 1: e0190498. 10.1371/journal.pone.0190498. 29338011 PMC5770016

[ece372424-bib-0093] Weindruch, R. , T. Kayo , C.‐K. Lee , and T. A. Prolla . 2001. “Microarray Profiling of Gene Expression in Aging and Its Alteration by Caloric Restriction in Mice.” Journal of Nutrition 131, no. 3: 918S–923S. 10.1093/jn/131.3.918S. 11238786

[ece372424-bib-0094] Westgate, A. J. , and A. J. Read . 2007. “Reproduction in Short‐Beaked Common Dolphins ( *Delphinus delphis* ) From the Western North Atlantic.” Marine Biology 150, no. 5: 1011–1024. 10.1007/s00227-006-0394-1.

[ece372424-bib-0096] Yagi, G. , H. Qi , K. Arai , et al. 2024. “Non‐Invasive Age Estimation Based on Faecal DNA Using Methylation‐Sensitive High‐Resolution Melting for Indo‐Pacific Bottlenose Dolphins.” Molecular Ecology Resources 24, no. 2: e13906. 10.1111/1755-0998.13906. 38041546

[ece372424-bib-0097] Zhang, P. 1993. “Model Selection via Multifold Cross Validation.” Annals of Statistics 21, no. 1: 299–313.

[ece372424-bib-0098] Zoller, J. A. , and S. Horvath . 2024. “MammalMethylClock R Package: Software for DNA Methylation‐Based Epigenetic Clocks in Mammals.” Bioinformatics 40, no. 5: btae280. 10.1093/bioinformatics/btae280. 38656974 PMC11091737

[ece372424-bib-0099] Zoller, J. A. , A. T. Lu , A. Haghani , S. Horvath , and T. Robeck . 2025. “Enhancing Epigenetic Aging Clocks in Cetaceans: Accurate Age Estimations in Small Endangered Delphinids, Killer Whales, Pilot Whales, Belugas, Humpbacks, and Bowhead Whales.” Scientific Reports 15, no. 1: 4048. 10.1038/s41598-025-86705-5. 39900928 PMC11791194

[ece372424-bib-0100] Zou, H. , and T. Hastie . 2005. “Regularization and Variable Selection via the Elastic Net.” Journal of the Royal Statistical Society, Series B: Statistical Methodology 67, no. 2: 301–320. 10.1111/j.1467-9868.2005.00503.x.

